# In silico mutational analysis and identification of stability centers in human interleukin-4

**DOI:** 10.22099/mbrc.2018.28855.1310

**Published:** 2018-06

**Authors:** Sandeep Saini, Chander Jyoti-Thakur, Varinder Kumar, Akshay Suhag, Niharika Jakhar

**Affiliations:** Department of Bioinformatics, G.G.D.S.D. College, Chandigarh, India

**Keywords:** IL-4; stability centre; mutagenesis; cytokine; bioinformatics

## Abstract

Interleukin-4 (IL-4) is a multifunctional cytokine that plays a critical role in apoptosis, differentiation and proliferation. The intensity of IL4 response depends upon binding to its receptor, IL-4R. The therapeutic efficiency of interleukins can be increased by generating structural mutants having greater stability. In the present work, attempts were made to increase the stability of human IL-4 using in-silico site directed mutagenesis. Different orthologous sequences of IL4 from *Pan troglodytes*,* Aotusnigriceps*, *Macacamulatta*, *Papiohamadryas*, *Chlorocebusaethiops*, *Vicugnapacos*, *Susscrofa *and *Homo sapiens* were aligned using Clustal Omega that revealed the conserved and non-conserved positions. For each non-conserved position, possible favorable and stabilizing mutations were found using CUPSAT with predicted ΔΔG (kcal/mol). The one with highest ΔΔG (kcal/mol) among all possible mutations, for each non-conserved position was selected and introduced manually in human IL-4 sequence resulting in multiple mutants of IL-4. Mutant proteins were modeled using structure of IL4 (PDB ID: 2B8U) as a template by SWISS MODEL. The mutants A49L and Q106T were identified to have stability centre using SCide. Molecular dynamics and docking analysis also confirmed the mutants stability and binding respectively. Mutants A49L and Q106T had -7.580079 kcal/mol and -39.418124 kcal/mol respectively lesser energy value than the wild type IL4. The result suggested that, the stability of human IL-4 has been increased by mutation.

## INTRODUCTION

Cytokines are group of small secreted proteins released by cells and known with different names like lymphokine (lymphocytes), monokine (monocytes), chemokine (chemotactic activities), and interleukin (leukocyte) depending upon the nature of secreting cells and activity [1]. These low molecular weight versatile proteins have played role in inflammation, immunity, differentiation and activation by acting on the cells that secrete them (autocrine action), on nearby cells (paracrine action), or in some instances on distant cells (endocrine action) [2]. Cytokines include interleukins (ILs), interferons (IFNs), growth factors, colony stimulating factors (CSFs), the tumors necrosis factors (TNF) and chemokines [3]. The cytokine to be discussed here are the interleukins, which are secreted proteins that act on leukocytes and other tissue targets such as kidney, intestine and liver etc. [4, 5], and have complex immunological functions including proliferation, migration, growth and differentiation. Whatever functions these proteins performed, the intensity of response and downstream signaling depends upon binding to their particular receptors present on targeted cells membrane [6-8].

Interleukin-4 (IL-4) is a multifunctional pleiotropic cytokine that play critical role in the proliferation, differentiation and apoptosis of many different cell types [9]. Primarily secreted by mast cells, Th2 cells, eosinophils and basophils [10], this cytokine shown to inhibit proliferation of renal carcinoma [11], act as stimulant for leukocyte survival [12], induces IgE class switching [13] and initiate protective immune responses against helminthes infection [14]. All the mentioned biological effects are initiated when IL4 binds to its receptor (IL-4R) present on the diverse array of cells like hematopoietic, endothelial, epithelial, muscle, fibroblast, hepatocytes and neuron [15]. IL-4R complex divided in to two types: type I and type II. Type I receptor complex consist of ligand binding IL-4R alpha chain (IL-4Rα) and a common gamma (γc) chain whereas in type II receptor γc chain is replaced by IL-13Rα1 to form receptor for interleukin-13 (IL-13), a functionally similar cytokine to IL-4 [9]. Binding of IL-4 to IL-4R induces dimerization of the chains that results in activation of Janus kinases (JAK1 and JAK3). The activated kinases phosphorylates tyrosine residues (Y573, Y603 and Y631) at IL-4Rα and provides binding site for STAT (Signal Transducers and Activators of Transcription), a transcription factor that induces cell response by activation of genes [9, 16].

Different cytokines including interleukins are shown to have therapeutic roles in critical diseases and are clinically tested and approved as drug for therapy [17-20]. Molecular engineering techniques such as PEGylation, fusion proteins, antibody complexes and mutagenesis are used for reducing toxicity and increasing half-life of therapeutics cytokines [3]. Structure-based cytokine engineering involving directed mutational strategies has opened new paradigm for use of cytokines both as agonist and antagonist [21]. 

In this article, our approach involves insilico mutagenesis for increasing stability centers in IL-4 that can have profound effect on binding to its receptor (IL-4R) and efficient downstream signaling. For achieving this, we used sequence alignment and molecular modeling approaches previously used for in silico analysis of different proteins [22-26].

## MATERIALS AND METHODS

**Data Retrieval: **Structural and sequence data of human wild type IL-4 was downloaded from PDB (Protein Data Bank), a database of experimentally (NMR or X-Ray crystallography) determined three-dimensional structures of protein [27]. PDB ID: 2B8U having resolution1.8Å was downloaded along with its FASTA sequence. IL-4 contains a single A chain having 129 amino acids dominantly forming the alpha helices.

**Orthologous analysis: **As the interleukins are constantly evolving [6], there is need to gather the sequence data from different species for the same interleukin to find out the conserved and non-conserved amino acids. IL-4 FASTA sequence retrieved from PDB was searched using BLAST-P [28] for obtaining orthologous; the search was constrained to mammals only.

**Multiple sequence alignment: **Multiply aligning the sequences of Orthologous gives insight in to conserved and non-conserved amino acids sites. Clustal Omega program was used for IL-4 Orthologous multiple sequence alignment. The program works on progressive alignment method and aligns the sequences globally by taking consensus of the pairwise alignment [29]. 

**Prediction of favorable and stabilizing mutations: **CUPSAT (Cologne University Protein Stability Analysis Tool) was used for the prediction of protein stability changes upon point mutations (single amino acid substitution). The program predicts the stability based on torsional angle potential, solvent accessibility and difference in free energy of unfolding between wild-type and mutant proteins [30]. Wild type IL-4 structure (2B8U) was used as input for finding the favorable and stabilizing mutations among non-conserved ‘:’ (colon) multiple alignment positions with rest of 19 amino acids. It requires the protein structure in Protein Data Bank format and the location of the residue to be mutated.

**Modeling of IL-4 mutants using SWISS-MODEL: **The most stable and favorable mutations obtained by CUPSAT for all the non-conserved positions is introduced into wild IL-4 sequence manually. The mutant variants were modeled using SWISS-MODEL [31] by taking 2B8U as template. All the mutant variants were having 99% sequence identity with template 2B8U because only one residue is substituted in each mutant.

**Identification of stability centers: **SCide (Supervisory Control Integrated Development Environment) is used to identify the stabilization centers of all modeled protein structures. Stabilization centers are the residues involved in cooperative long-range contacts in a protein, which are important in maintaining the stability of a protein three dimensional structures [32]. All the optimized modeled mutants were checked for stability centers through SCide. 

**Molecular dynamics studies: **Discovery studio [33] was used to apply CHARMM [34] force field to all the IL-4mutants structures in order to optimize the structures. Hyperchem **[**35**] **was used to minimize and calculate the overall energy of IL-4 mutants and wild type structures using two different algorithms of energy minimization, steepest descent [36] and conjugate gradient [37] by customize iterations to 100 times.

**Docking analysis:** The binding energy of receptor (IL-4R) and ligand was calculated using HEX 8.0 [38], a protein-protein docking tool. PDB ID: 1IAR was downloaded from protein data bank; this structural file is the complex of IL-4 and receptor alpha chain. For the docking, separate receptor and ligand files were prepared using pymol. Water molecules were also removed from crystallography structure. The IL-4 (separated from complex 1IAR) and modeled mutants (A49L, Q106T) were treated as ligand and receptor alpha chain as receptor for calculation of binding energies.

## RESULTS

**Figure 1:**
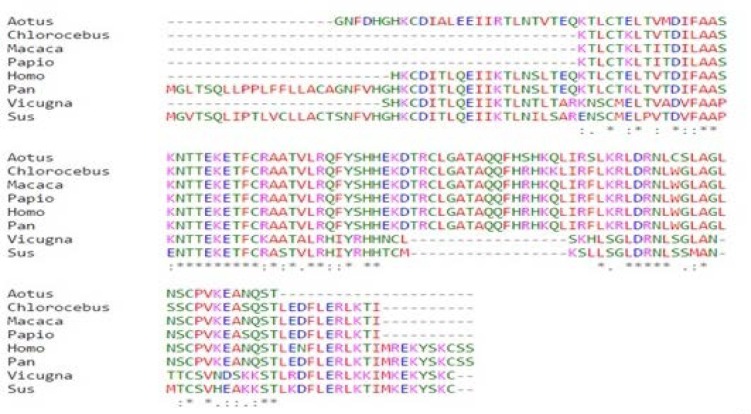
Clustal Omega result. Showing the conserved (*) and non-conserved (:) amino acid positions

In silico analysis of proteins provide a means of rapidly analyzing the sequence and structural aspects of growing number of proteins in post-genomic era [39, 40]. Using a well defined strategy for mutational analysis, we analysed IL-4 for its structural stability by finding the stability centers in it. The results obtained through computational tools are discussed in chronological order. Human wild type IL-4 sequence searching using BLAST-P among mammals resulted inretrieval of IL-4 orthologous in *Pan* *troglodytes*, *Aotusnigriceps*, *Macacamulatta*, *Papiohamadryas*, *Chlorocebusaethiops*, *Vicugnapacosand* *Susscrofawith* varying sequence percent identity in the range of 98-54 %. All the wild type sequences of IL-4 from above mentioned species including human showed conserved ‘*’(asterisk) and non-conserved ‘:’ (colon) amino acids positions after analysis with Clustal omega program of multiple sequence alignment. Among the substitution, only ‘:’ positions which resulted by substitution with similar physiochemical properties amino acids were considered for further study and ‘.’ (period) that represents change with weakly similar amino acids residues were left out [41, 42]. Following are the 15 colon positions with respective amino acid observed in human IL-4: 21K, 26E, 29V, 32I, 33F, 37K, 47R, 49A, 54Q, 55F, 93L, 102K, 103E, 104A and 106Q as shown in Figure 1. All these positions can have the tendency to mutate in human IL-4 as orthologous sequences showed substitution in same positions [43]. 

CUPSAT mutational analysis results provide the amino acid residues with the favorable and stabilizing mutation with predicted ΔΔG (kcal/mol). It consists information about mutation site, its structural features and comprehensive information about changes in protein stability for 19 possible substitutions for a specific amino acid position. A total of 11 stable and favorable mutations were found using CUPSAT and for 4 positions (V29, F33, K37, L93) no stable substitutions were found as shown in Table 1. 

**Table1:** The list of stabilizing and favorable mutations found by CUPSAT and selected mutation based on highest ΔΔG (kcal/mol)

**S.NO.**	**Residue present in wild type IL-4**	**Stabilizing and favorable** **Mutations**	**Selected mutation**
1	LYS21	SER, THR, ASP	THR
2	GLU26	LEU, TRP, SER, THR, LYS, ASN, ASP, HIS	SER
3	VAL29	-	-
4	ILE32	PRO	PRO
5	PHE33	-	-
6	LYS37	-	-
7	ARG47	LEU, ILE, GLU, MET, PHE, LYS, TYR	ILE
8	ALA49	LEU, MET	LEU
9	GLN54	LEU, TRP, GLU, ARG	TRP
10	PHE55	TRP	TRP
11	LEU93	-	-
12	LYS102	THR, GLN, ASN, ARG, HIS	THR
13	GLU103	PRO	PRO
14	ALA104	VAL, ILE, THR, GLN, LYS, HIS	HIS
15	GLN106	VAL, LEU, MET, THR, TYR	THR

**Figure 2:**
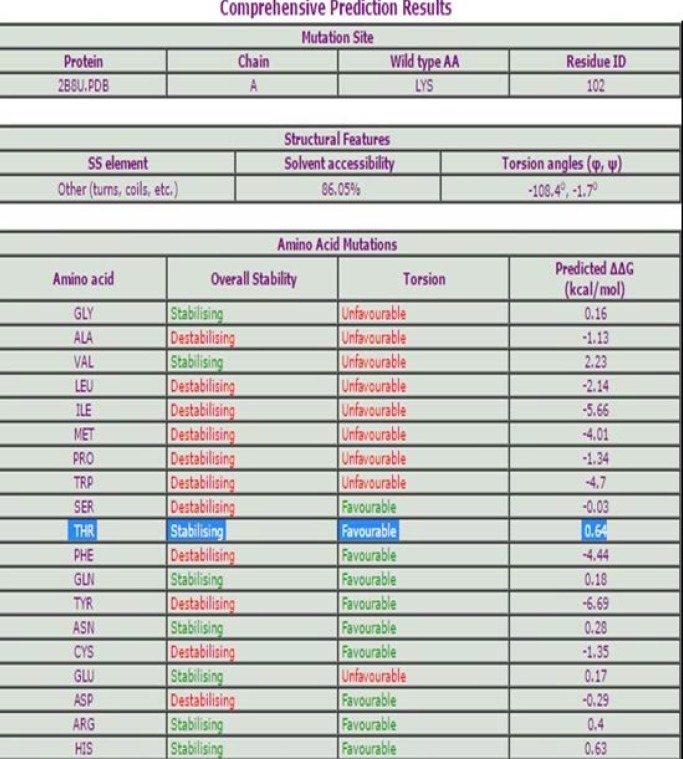
CUPSAT analysis result. Showing the stabilizing and favorable mutations for Lys102

**Figure 3:**
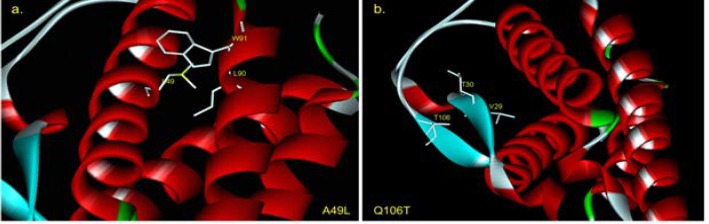
SCide result. Showing the stability centers residues interacting with mutants.3a.) mutant A49L interacting with L90 and W91. 3b) mutant Q106T interacting with V29 and T30

The favorable and stabilizing mutation with highest ΔΔG (kcal/mol) among all possible mutations were selected. For instance, possible mutations for Lysine in wild type at 102 are THR, GLN, ASN, ARG, HIS. But we selected amino acid with highest ΔΔG (kcal/mol) among all possible mutations i.e. THR (Fig. 2) .SWISS MODEL provides 11 modeled mutants protein structures with desired stable mutations, selected after CUPSAT analysis. Out of total 11 mutants, SCide identified stabilization centers in mutants A49L and Q106T as shown in Table 2. The mutant A49L residue interacts with L90, W91 and Q106T interacts with V29 and T30 (Fig. 3). All other mutants were not found to have any stabilization centers.

**Table 2:** List of stabilization center obtained by SCide

**S.No.**	**Mutants**	**Stabilization Center**
1	K21T	------
2	E26S	------
3	132P	------
4	R47I	------
5	**A49L**	**PRESENT(L90,W91)**
6	Q54W	------
7	F55W	------
8	K102T	------
9	E103P	------
10	A104H	------
11	**Q106T**	**PRESENT(V29,T30)**

Molecular dynamics studies provide optimized structures after application of force field. The energy minimization algorithms gave minimum energy of three dimensional structures of all mutants and wild IL-4 (2B8U) as shown in Table 3. Comparison of energies of wild and mutants (A49L and Q106T) that were found to have stabilization centers showed decrease in energies for mutants. The mutant A49L has -7.580079 kcal/mol lesser energy than wild IL-4 by steepest descent algorithm. The energy of the mutant Q106T has significant decrease of -39.418124 kcal/mol and -1938.748829 kcal/mol by steepest descent and conjugate gradient algorithms respectively. The approach of energy minimization proved to be significant while comparing the stabilities of mutants and wild type protein structures. A protein structure with lesser energy can be more stable than others [44-47].

**Table 3:** Minimum energies of wild type and mutants protein structures

**S.NO.**	**Protein type**	**Steepest Descent (kcal/mol)**	**Conjugate gradient (kcal/mol)**
1	2B8U(wild type)	-719.475296	-991.094145
2	**Q106T**	**-758.893420**	**-992.654684**
3	**A49L**	**-727.055375**	**-981.798658**
4	A104H	-764.236576	-994.518668
5	E26S	-772.544228	-996.045970
6	E103P	-693.857297	-953.770159
7	F55W	-716.39280	-987.615785
8	I32P	-748.010414	-982.546200
9	K21T	-760.211250	-1002.348249
10	K102T	-725.256304	-968.994311
11	Q54W	-753.569414	-992.150766
12	R47I	-710.624625	-968.129800

Docking results obtained through HEX for calculation of binding energies was also significant for mutants (A49L and Q106T). The binding energies of A49L and Q106T with receptor alpha chain were found to be lesser compared to wild IL-4 as shown in Table 4. Even the RMS (Root Mean Square) deviation values are acceptable for mutants. So there is no changed in conformation of mutants from its wild type structure after substitutions, which are desired for it to be biologically active [48-50].

**Table 4:** HEX Result

**S.No.**	**Ligand**	**Binding Energy with IL4-alpha Receptor**	**RMS Deviation**
1	IL-4(Wild type)	-708.86	-1.00
2	Q106T	-731.58	-1.00
3	A49L	-721.51	-1.00

## DISCUSSION

Site directed mutagenesis often used for increasing the stability of protein [51] and requires well established protocols for insertion, deletion and substitution of point mutations in desired sequences of interest [52]. But with the advancement of computational strategies the stability of engineered protein can be predicted with ease and proved to be significant for designing *in vitro* and *in vivo* experimental protocols [53]. Relationship between mutational, structural and functional behavior of protein has been established many times [54, 55].

Enhancement in the binding affinity of different interleukins (IL-2, IL-3 and IL-5) to their corresponding receptors (IL-2Rα, IL-3Rα and IL-5Rα) by mutations has already proven to be significant [56-58]. In addition, different other cytokines such as interferon [59], growth factor [60], colony stimulating factor [23], tumors necrosis factor [61], chemokine [62] and other diverse array of proteins [63-66], whose effects depends upon binding to cell surface receptors of their target cell was analyzed computationally for prediction of favorable and unfavorable mutations.

Acting as immunomodulator, IL-4 has many important roles like class switching of antibody IgE, anti-inflammatory agent and inhibition of tumor in animal model and in-vitro studies [67]. Previous clinical studies of IL-4 therapy in human patients resulted in some serious adverse effects like increase in platelet counts, cardiac toxicity and gastrointestinal ulceration etc. [68, 69]. Mutational studies on receptor IL-4R have been reported previously [70, 71] but lack of mutational studies on its ligand IL-4 reduce its therapeutics capabilities. The issues like cytokines toxicity, short half life and their pleiotropic nature some time involve in occurrence of adverse effects during therapy [72]. Structural stability of therapeutic proteins can minimize the adverse effects [73].

Here, we used combination of evolutionary and mutational approach to find amino acid residues positions that can have the tendency to mutate i.e. non-conserved sites. Further, all the sites were checked for the favorable and stable mutations both structurally and energetically. The intent was to identify easily mutable positions and residues that can prove to be stable structurally because the effect of therapy depends on structural stability of the drug molecule. The two modeled mutants (A49L, Q106T) are found to have lesser energy compared to wild type IL-4. In addition, only these two mutants were also found to have stabilization centers that can further stabilize the structure by long range interactions. Results are further supported by calculating the binding energies of these two mutants that proved to be significant compared to wild IL-4. The identified stable mutations can be tried to introduce in-vitro for validating the in-silico work and making more safe and effective therapeutic IL-4 drug molecule.

## Conflict of Interest:

The authors declare that they have no conflict of interest.
